# Can parent-infant observation predict later childhood psychopathology: a systematic review

**DOI:** 10.1192/bjo.2021.155

**Published:** 2021-06-18

**Authors:** Elena McAndie, Lucy Thompson, Philip Wilson

**Affiliations:** 1 CAMHS, NHS Grampian; 2 Centre for Rural Health, University of Aberdeen

## Abstract

**Aims:**

Difficulties in parent-child interaction are easily observed and are a potential target for early intervention. This study aimed to assess the utility of observation of parent-child interaction in the first year of life in identifying children at risk of developing later psychopathology, using a rigorous systematic review method.

**Method:**

EMBASE, CINAHL, PsycINFO, MIDIRS, MEDLINE and Cochrane Library databases were searched using MeSH terms and keywords, and reference lists screened. Two authors independently reviewed papers for inclusion and completed data extraction. All peer reviewed papers studying the association between an independent observation of parent-child interaction and later childhood psychopathology in community-based samples were included. Studies based on ‘high risk’ samples (studies exclusively examining cohorts with a sibling or parent with a mental illness or studies of low birth weight or premature infants and those with other physical comorbidities) were excluded. Results were synthesised qualitatively due to high heterogeneity.

**Result:**

18,226 papers were identified, nine were included in this study. Childhood psychopathology was associated with fewer positive parent-infant interactions, lower parent vocalisation frequency and lower levels of adult speech and activity. Maternal sensitivity was inversely related to separation anxiety and oppositional defiant/conduct disorders were associated with lower shared look rates. Disruptive behaviour disorders were associated with higher frequency of child vocalisation. Pervasive developmental disorders were associated with ‘abnormal’ maternal infant interactions, as assessed by community health nurses using a standardised measure.

**Conclusion:**

Included studies reported small samples, and several of these samples overlapped. Some studies were of poor quality, but were included due to a paucity of available data. The findings may therefore have limited generalisability. Difficulties in parent-child interaction are easily observed and assessments could be made by non-specialists such as health visitors or general practitioners. Such difficulties may be an early indicator of later childhood psychopathology. Childhood psychiatric diagnoses (with the exception of Autistic Spectrum Disorders) appear associated with level of maternal activity (vocalisation, physical activity, positive parenting and shared attention). Assessments may identify at-risk families for early intervention, but further work is required to develop and validate reliable methods for risk stratification in community-based practice.

